# Potential novel proteomic biomarkers for diagnosis of vertebral osteomyelitis identified using an immunomics protein array technique: Two cases reports

**DOI:** 10.1097/MD.0000000000022852

**Published:** 2020-10-23

**Authors:** Chang-Hua Chen, Ing-Lin Chang, Shu-Hui Wang, Hua-Cheng Yen, Jen-Shiou Lin, Shou-Chen Lo, Chieh-Chen Huang

**Affiliations:** a Division of Infectious Disease, Department of Internal Medicine; b Center for Infection Prevention and Control, Changhua Christian Hospital, Changhua; c Ph.D. Program in Translational Medicine, National Chung Hsing University; d Rong Hsing Research Center For Translational Medicine, National Chung Hsing University, Taichung City; e Department of Orthopedic Surgery; f Division of Critical Care Medicine; g Department of Neurosurgery; h Department of Laboratory Medicine, Changhua Christian Hospital, Changhua; i Department of Life Sciences; j PhD Program in Medical Biotechnology, National Chung Hsing University, Taichung City, Taiwan.

**Keywords:** aldolase, Case Report., diagnostic biomarkers, fructose-bisphosphate A, keratin 8, pterin-4 alpha-carbinolamine dehydratase 1, Tyrosine 3- Monooxygenase/Tryptophan activation protein gamma, Vertebral Osteomyelitis

## Abstract

**Rationale:**

Although vertebral osteomyelitis (VO) is commonly associated with high morbidity and high recurrence rate, effective diagnostic and prognostic biomarkers of VO are still lacking.

**Patients concerns:**

Case 1: a 60-year-old male had had upper back pain for 3 days. Case 2: a 71-year-old female presented upper back pain for 2 days.

**Diagnoses:**

Based on physical examination and findings of magnetic resonance imaging and findings by matrix-assisted laser desorption ionization-time of flight mass spectrometry, they were diagnosed with *Staphylococcus aureus* VO.

**Interventions:**

Using Sengenics Immunome^TM^ Protein Array by analyzing autoantibodies in both VO patients, potential biomarkers of VO were explored.

**Outcomes:**

Four subjects with more than 1600 antigens screened while the results showed that 14-3-3 protein gamma, pterin-4-alpha-carbinolamine dehydratase, fructose-bisphosphate aldolase *A*, and keratin type II cytoskeletal 8 were highly differentially expressed among VO and controls. Relevant auto-antibody profiles were discovered after intra-group and inter-group comparison, and based on functional rationality, an adapter protein 14-3-3 protein gamma, and pterin-4-alpha-carbinolamine dehydratase that involved in tetrahydrobiopterin biosynthesis, might serve as valuable diagnostic biomarkers.

**Lessons:**

This pilot study on 4 subjects with more than 1600 antigens screened on the Sengenics Immunome protein array provided a general outlook on autoantibody biomarker profiles of VO subjects. Future large-scale trials with longer follow-up times are warranted.

## 1 Introduction

Vertebral osteomyelitis (VO) is an infection of the vertebrae, contiguous intervertebral disc space, and the paravertebral space.^[1,2]^ VO is commonly associated with high morbidity, expenditure, and recurrence.^[3]^ Delayed diagnosis leading to disabling morbidity is common, and functional disabilities occur in 25% to 50% of cases.^[4]^ Early identification of VO patients is critical to ensure accurate clinical decisions and improved prognosis. Currently, the gold standard of VO diagnosis is magnetic resonance imaging (MRI).^[4]^ In a preliminary study, we focused on the clinical presentation of VO in Taiwan.^[5]^ However, the clinical diagnosis of VO remains challenging since it lacks sensitive and accurate markers for detection, and does not have a convenient gold standard for investigation. Furthermore, it is still unclear what factors might determine the relatively high morbidity and mortality, and which of these is the key determinant. Therefore, diagnostic judgments require further studies to identify prognostic biomarkers that can predict potential complications in VO patients.

At present, there are no reliable biomarkers that could identify VO patients with bacteremia in the clinic. Recent studies have identified several VO biomarkers, which are listed in Supplemental Digital Content (Appendix 1). Total white cell count, erythrocyte sedimentation rate (ESR),^[4]^ C-reactive protein (CRP),^[6]^ and procalcitonin (PCT)^[7]^ are routinely used in the diagnosis of these infections, but no specific laboratory test exists, with the exception of the isolation of pathogenic organisms from the bone or synovial fluid.^[8]^ CRP and ESR are currently used in all patients suspected of having VO; however, they lack the accuracy to differentiate between patients with and without VO.^[4]^ Anti-glucosaminidase IgG,^[9]^ iron-regulated surface determinant protein B,^[10]^ CCL11/eotaxin,^[11]^ interleukin-6,^[11]^ S100 calcium binding protein A11, E-selectin, and matrix metalloproteinase-1^[12]^ have been reported as potential biomarkers^[13]^; yet, they have not been used for the clinical diagnosis of VO. A review of the literature, employing a data mining approach using Open Targets Platform (https://www.targetvalidation.org/),^[14]^ presented a list of VO-associated antigens showing elevated autoantibody responses^[14]^; the results are shown in Supplemental Digital Content (Appendix 2, which lists open target database). Finding diagnostic biomarkers that could reduce the delayed diagnosis time from VO onset, allow early initiation of the correct treatment, and have the potential to reduce overall mortality would be beneficial. However, there is currently a deficiency in available diagnostic VO biomarkers.

Development of biomarkers with high predictive value for infectious diseases is 1 of the goals for precision medicine. Early diagnosis, patient stratification, and predictive treatment of infectious diseases using biomarkers can significantly improve treatment effectiveness and the survival rate of patients, especially in the case of acute and difficult infectious diseases.^[4]^ The Immunome^TM^ Protein Array utilizes an immunomics technique employing a biotin carboxyl carrier protein domain affinity tag to promote fusion of post-translational biotinylated proteins to an array surface. This surface includes 1,631 proteins that serve important functions in the human immune response, including kinases and transcription factors.

We described 2 VO cases. Both cases are difficult to diagnose VO at the critical moment because there is lack of accurate VO biomarkers. And we designed this pilot study to identify potential VO biomarkers, those immune-related proteins expressed in VO patients with different clinical condition by Immunome^TM^ profiling. The Immunome^TM^ Protein Array also allows for the detection of autoantibodies in patient serum samples, making it an effective tool for biomarker discovery. Therefore, this study aimed to determine the differential expression levels of immune-related proteins identified in patients with different VO classifications, in order to identify proteomic biomarkers in VO patients. Furthermore, it was designed to provide insights into potential pathways of VO progression and enable the development of novel treatment strategies and appropriate clinical management of VO patients.

## 2 Methodology

In order to detecting biomarkers of VO, we designed the approach method as shown in Supplemental Digital Content (Appendix 3). In brief, 4 subjects, including 2 VO subjects, 1 negative control subject (bone degeneration disease patient without infection), and 1 healthy control subject, were enrolled between July 1, 2018 to December 31, 2018. Of the 4 subjects, including 2 patients were analyzed (Supplemental Digital Content (Appendix 4, which summaries the clinical data from 4 subjects, including 2 patient information)) while 2 had diabetes mellitus, 2 were hypertensive, and 1 had end-stage renal disease. None of the subjects had a predisposing traumatic condition. As for the initial signs and symptoms before admission, 3 had a painful sensation over the lesion site, and 2 had fever. The initial laboratory data showed that 2 had leucocytosis, 2 had elevated ESR, 1 had elevated CRP level, and 1 had elevated PCT level. Initial blood culture showed the presence of *Staphylococcus aureus* in 2 subjects. The initial MRI images were compatible with osteomyelitis in 2 subjects. All subjects survived. Previous potential biomarkers of VO, including ESR, CRP, and PCT were found to be partially significant.

### 2.1 Case 1 presentation

A 60-year-old male, who had had diabetes mellitus, hypertension and end stage renal disease, was sent to our emergency department because of upper back pain for 3 days, and he presented with episodic fever and general malaise. He was admitted for further evaluation and management. He denied any traumatic injury and acupuncture and invasive therapeutics. At admission Day, his vital sign showed a blood pressure of 120/80 mmHg, body temperature 37.6°C, respiratory rate 24/min and heart beat 88/min. Physical examination and important clinical findings showed the painful sensation over intra-scapular area and there were no significant neurological deficit. The initial laboratory data revealed white cell count, 36700/mm^3^; ESR, 32 mm/hr; CRP, 3.48 mg/L and PCT, 0.6 ng/mL. The magnetic resonance imaging (MRI) of the spine showed that abnormal collection with T1 and T2 high signal intensity at the C4 to T2 level, around the vertebrae and suspected connection with C7/T1 disk space and adjacent C7-T1 anterior epidural space involvement are found. The C7-T1 diskitis with VO was impressed. *Staphylococcus aureus* (2/2 sets) was identified by using matrix-assisted laser desorption ionization-time of flight mass spectrometry (bioMerieux, Hazlewood, Mo.) on 3^th^ admission day, and it was oxacillin resistant strain according to the susceptibility test. Oxacillin resistant *S. aureus* VO was suspected, and parenteral vancomycin 500 mg plus daptomycin 300 mg (6 mg/kg) every other day after dialysis was prescribed. He received the 42-days combination regimens without adverse and unanticipated events. The serial follow-up laboratory data normalized. He was discharged on 45^th^ admission days and was follow-up at outpatient department where he recovered well.

### 2.2 Case 2 presentation

A 71-year-old healthy female visited to our emergency department because of upper back pain for 2 days, and she presented with fever. She was admitted for further evaluation and management. She denied any traumatic injury and acupuncture and invasive therapeutics. At admission Day, her vital sign showed a blood pressure of 140/90 mm Hg, body temperature 38.2°C, respiratory rate 26/min and heart beat 110/min. Physical examination and important clinical findings showed the painful sensation over middle part of T spine area and there were no significant neurological deficit. The initial laboratory data revealed white cell count, 9300/mm^3^; ESR, 86 mm/h; CRP, 0.28 mg/L and PCT, 0.03 ng/mL. The MRI of the spine showed that abnormal signal intensity in the T8–9 vertebrae. The T8–9 VO was impressed. *S aureus* (2/2 sets) was identified by using matrix-assisted laser desorption ionization-time of flight mass spectrometry (bioMerieux, Hazlewood, Mo.) on 3^th^ admission day, and it was oxacillin susceptible strain according to the susceptibility test. Oxacillin susceptible *S. aureus* VO was suspected, and parenteral oxacillin 2000 mg every 4 hours was prescribed. She received the 42-days combination regimens without adverse and unanticipated events. The serial follow-up laboratory data normalized. She was discharged on 44^th^ admission days and was follow-up at outpatient department where she recovered well.

The penetrance fold change-based method was performed for the following 2 sets of analysis. First, analysis of VO plasma versus non-VO control (bone-disease patient without infection) plasma was conducted; Supplemental Digital Content (Appendix 5) shows the top 10 antigens with high autoantibody titers identified from the analysis. Penetrance fold changes between VO plasma and non-VO control were as follows: geranylgeranyl pyrophosphate synthase (GGPS1), 20.78; replication protein A 32 kDa subunit (RPA2), 18.884; Sjoegren syndrome nuclear autoantigen 1 (SSNA1), 17.281; ornithine decarboxylase (ODC1), 14.832; keratin, type II cytoskeletal 8 (KRT8), 14.481; alpha-crystallin B chain (CRYAB), 14.137; keratin, type I cytoskeletal 19 (KRT19), 14.026; fructose-bisphosphate aldolase A (ALDOA), 13.233; cysteine-rich secretory protein 2 (CRISP2), 12.781; and cAMP-dependent protein kinase type I-alpha regulatory subunit (PRKAR1A), 12.661. Second, analysis of VO plasma compared to healthy control (healthy person) plasma was performed; Supplemental Digital Content (Appendix 6) shows the top 10 antigens with high autoantibody titers identified from this analysis. Penetrance fold changes between VO plasma and the healthy control were as follows: zinc finger C4H2 domain-containing protein (ZC4H2), 11.391; KRT8, 8.819; transforming acidic coiled-coil-containing protein 1 (TACC1), 8.61; ODC1, 6.949; MOB kinase activator 3A (MOB3A), 6.291; pterin-4-alpha-carbinolamine dehydratase (PCBD1), 6.286; ALDOA, 6.027; 14-3-3 protein gamma (YWHAG), 5.998; thymidine kinase, cytosolic (TK1), 5.752; and DnaJ homolog subfamily B member 1 (DNAJB1), 5.685.

**Figure 1:**
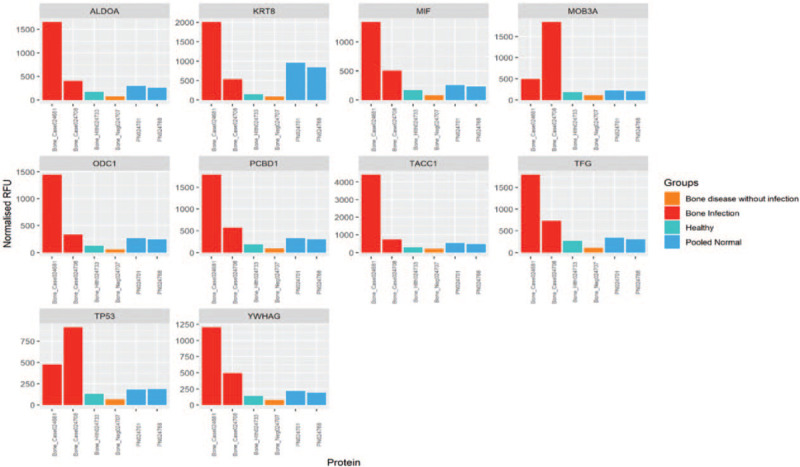
Autoantibody profile across VO, non-VO control, healthy control and pooled normal samples for the most clinically significant, common biomarkers identified based on penetrance fold change analysis between VO vs non-VO control and VO vs healthy control, separately. VO = vertebral osteomyelitis. Bone_Case024681 and Bone_Case024408 are 2 VO patient samples; Bone_Health024733 is 1 healthy control sample; Bone_Neg024707 is 1 non-VO patient sample; PN024701 and PN024768 are 2 pooled normal sera. VO = vertebral osteomyelitis.

**Figure 2:**
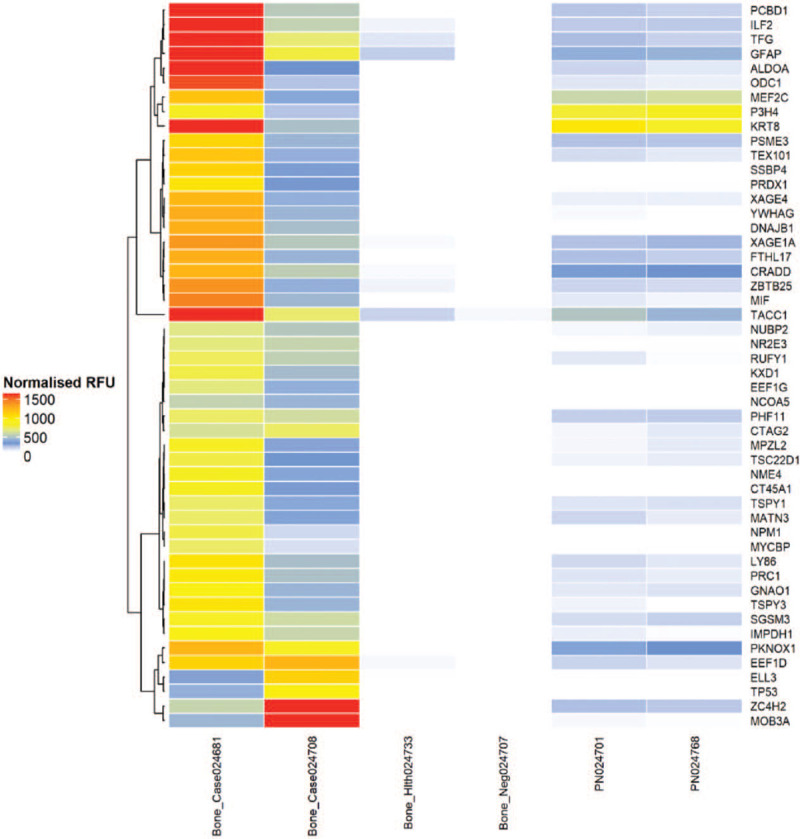
Unsupervised clustering of individual fold change profiles for 50 biomarkers across 2 VO patient samples, 1 non-VO control, 1 healthy control and 2 pooled normal sera. The clustering was performed based on Ward's method with distance calculated based on Euclidean distance. VO = vertebral osteomyelitis. Bone_Case024681 and Bone_Case024408 are 2 VO patient samples; Bone_Health024733 is 1 healthy control sample; Bone_Neg024707 is 1 non-VO patient sample; PN024701 and PN024768 are 2 pooled normal sera. VO = vertebral osteomyelitis.

An unsupervised clustering was performed for the following biomarkers across patients, controls, and pooled normal samples using a hierarchical clustering method with distance calculated based on Euclidean distance. The heatmap shown in Figures 1 and 2 were plotted using ComplexHeatmap v 1.20.0 package in Bioconductor.

A comparison of autoantibody profiles among VO, non-VO control, healthy control, and pooled normal samples for the most clinically significant common biomarkers identified based on penetrance fold change analysis is shown in Figure 1. The levels of all antigens were elevated in the 2 VO samples. Individually, the levels of GGPS1, RPA2, SSNA1, KRT8, CRYAB, KRT19, ALDOA, CRISP2, PRKAR1A, ALDOA, KRT8, macrophage migration inhibitory factor (MIF), ODC1, PCBD1, TACC1, TGF, and YWHAG were elevated in subject 1; and those of MOB kinase activator 3A (MOB3A) and EKC/KEOPS complex subunit cellular tumor antigen p53RK were elevated in subject 2.

Figure 2 shows the 50 most common biomarkers across the VO samples, non-VO control, healthy control, and pooled normal sera, based on the penetrance fold change between VO and the negative control, and VO and the healthy control.

Clustering was performed according to Ward's method, and the Euclidean distance was calculated. Unsupervised clustering of individual fold change profiles for the 50 biomarkers among VO, non-VO control, healthy control, and pooled normal samples is shown in Figure 2. The levels of all antigens were elevated among the 2 VO samples. Individually, PCBD1, interleukin enhancer-binding factor 2 (ILF2), protein TFG (TFG), GFAP, ALDOA, ODC1, KRT8, and transforming acidic coiled-coil-containing protein 1 (TACC1) showed high normalized relative fluorescence units (RFU) in subject 1; and ZC4H2 and MOB3A showed high RFU in subject 2.

In the comparison between the VO patients and the non-VO control, KRT8 was selected because the penetrance fold change was 14.481, and the penetrance percentage frequency was 100%. ALDOA was selected because the penetrance fold change between VO patients and the non-VO control was 13.233 and the penetrance percentage frequency was 100%. In addition, YWHAG showed a penetrance fold change of 5.998 after comparing VO patients and the healthy control group, and a penetrance percentage frequency of 100%. PCBD1 showed a penetrance fold change of 6.286 between VO patients and healthy controls, and a penetrance percentage frequency of 100%.

## 3 Discussion

This research explored biomarkers of VO using the Sengenics Immunome^TM^ Protein Array platform. We identified 2 distinct and novel biomarkers in this preliminary study conducted in Taiwan. There were also several other major findings. First, partial significance was found for known potential biomarkers of VO, including ESR, CRP, and PCT. Second, the following target antigens showed high penetrance fold change in a comparison between VO plasma and non-VO control plasma: GGPS1, RPA2, SSNA1, ODC1, KRT8, CRYAB, KRT19, ALDOA, CRISP2, and PRKAR1A. In addition, in an analysis comparing VO plasma and healthy control plasma, the following target antigens showed high penetrance fold change: ZC4H2 KRT8, TACC1, ODC1, MOB3A, PCBD1, ALDOA, YWHAG, TK1, and DNAJB1. Third, the top ten identified antigens obtained upon comparing the autoantibody profiles among VO, non-VO control, healthy control, and pooled normal samples were GGPS1, RPA2, SSNA1, KRT8, CRYAB, KRT19, ALDOA, CRISP2, PRKAR1A, ALDOA, KRT8, MIF, ODC1, PCBD1, TACC1, TGF, YWHAG, MOB3A, and cellular tumor antigen p53. Fourth, PCBD1, ILF, TFG, GFAP, ALDOA, ODC1, KRT8, TACC1, ZC4H2 and MOB3A were identified as the top ten antigens throughout the analysis. Finally, YWHAG and PCBD1 were identified as potentially clinically significant as diagnostic biomarkers of VO after intersecting at relevant auto-antibody profiles after intra-group and inter-group comparison and interpreting based on functional rationality. However, further study is needed in order to validate these novel biomarkers. Generally, ESR,^[5]^ CRP,^[10]^ or PCT^[6]^ cannot be used to diagnose VO patients. This study showed that previously reported potential biomarkers of VO, including ESR, CRP, and PCT were partially significant. Yet, these findings are important for identifying clinically significant common diagnostic biomarkers of VO, and the combination of biomarkers with diagnostic and prognostic potential in a defined VO condition can improve the overall diagnostic accuracy rate. This study aimed to discover the most significant autoantibody biomarkers of VO. The proposed functional annotations of the top 10 antigens from the Immunome protein microarray platform, with significant autoantibody responses in VO vs non-VO control samples, are listed in Supplemental Digital Content (Appendix 3). The comparison of their annotations for VO patient samples vs healthy control samples is listed in Supplemental Digital Content (Appendix 7). Out of all of the significant autoantibody biomarkers listed for VO, the following biomarkers may have clinical relevance.

YWHAG displayed a penetrance fold change of 5.998 between VO patients and the negative control and a penetrance percentage frequency of 100%. After employing a data mining functional approach using Universal Protein Resource (UniProt) Platform (https://www.uniprot.org/),^[15]^ YWHAG was discovered to be an adapter protein to regulate both general and specialized signaling pathways. YWHAG binds to many partners, often by recognizing a phosphoserine or phosphothreonine motif. Further, YWHAG binding usually creates the modulation of the activity of its binding partner. Chu et al. reported for the first time that the expression level of miR-222 was reduced in osteosarcoma (OS) tissues as well as in OS cell lines.^[16]^ Furthermore, miR-222 can inhibit cell proliferation and invasion via down-regulating YWHAG. These data suggest a potential target for the biological treatment of OS.^[16]^

The second potential biomarkers with clinical relevance in VO is PCBD1, with a penetrance fold change of 6.286 between VO patients and healthy controls and a penetrance percentage frequency of 100%. PCBD1 was found to be involved in tetrahydrobiopterin biosynthesis, and it seems to both prevent the formation of 7-pterins and accelerate the formation of quinonoid-BH2.^[15]^ PCBD1 plays the role of a coactivator for HNF1A-dependent transcription. PCBD1 regulates the dimerization of homeodomain protein HNF1A and enhances its transcriptional activity. Herzlieb et al.^[17]^ illustrated that IGF-I treatment of U-2 OS cells both inhibits the induced programmed cell death (PCD) and increases the cell count, effects which are blocked by addition of IGF-I receptor antibodies. Herzlieb et al. also hypothesized that IGF-I affects cells via 2 pathways: by promoting proliferative responses and by suppressing PCD. The differential responses of PCD and cell number to caspase 1 inhibitors suggest the existence of an independent control system for these processes.^[17]^

The third potential biomarker is ALDOA, with a penetrance fold change of 13.223 between VO patients and non-VO control and a penetrance percentage frequency of 100%. Between VO patients and the healthy control group the penetrance fold change was 6.027 and the penetrance percentage frequency was 100%. It was found that ALDOA plays a key role in glycolysis and gluconeogenesis.^[15]^ In addition, it may also function as a scaffolding protein. The biomarker points resistance to cisplatin for osteosarcoma and proposes for the early and accurate use of cisplatin to treat osteosarcoma. Zhang et al. described that ALDOA and PGK1 might become appropriate biomarkers for treating osteosarcoma with cisplatin.^[18]^

The fourth potential biomarker is KRT8, with a penetrance fold change of 14.481 between VO patients and the non-VO control group and a penetrance percentage frequency of 100%. Between VO patients and the healthy control group the penetrance fold change was 8.819 and the penetrance percentage frequency was 100%. It has been reported that KRT8 can link the contract apparatus to dystrophin at the striated muscle.^[15]^ Le Henaff et al. described that cystic fibrosis patients show low bone mass and alterations of bone structure,^[19]^ and mice bringing the F508del genetic mutation in the cystic fibrosis conductance regulator (*Cftr*) gene present decreased bone formation and reduced bone mass.^[19]^ The KRT8 could cause to correct osteoblast dysfunctions, to alter bone formation. And osteopenia in *F508del-Cftr* mice provides the KRT8*-*F508del-CFTR reaction to reform the abnormal bone structure in cystic fibrosis.^[19]^ In addition, the potential proteins associated with VO disease showing elevated autoantibody responses were reviewed based on literature and a data mining approach using Open Targets Platform (https://www.targetvalidation.org/)^[14]^ as well as UniProt Platform (https://www.uniprot.org/).^[15]^ The results are shown in Supplemental Digital Content (Appendix 8, which lists the disease associations of biomarkers identified across 4 subjects. Data was obtained from Open Targets platform.). Furthermore, Supplemental Digital Content (Appendix 9) lists annotations of biomarkers of bone diseases, including VO. Other biomarkers were observed which might play a significant role in immune responses related to VO. The autoantibody profiles of each case will gain unique significance when associated with the pathophysiology of the patients. This will help to identify potentially sustainable biomarkers that can assist with VO diagnosis. After discovering relevant autoantibody profiles and performing intra-group and inter-group comparisons, as well as interpreting them based on functional rationality, YWHAG and PCBD1 in particular showed potential as valuable diagnostic biomarkers of VO.

This pilot study on 4 subjects with more than 1600 antigens screened on the Sengenics Immunome protein array provided a general outlook on autoantibody biomarker profiles of VO subjects. YWHAG, and PCBD1 in particular might serve as valuable diagnostic biomarkers. Therefore, further research including a large-scale autoantibody profiling study to identify biomarkers with diagnostic and prognostic potential in a defined VO condition is warranted to validate these novel biomarkers.

## Acknowledgments

This research project would not have been possible without the support of many people. The authors wish to express their gratitude to the staffs of the Department of Neurosurgery, Orthopedic Surgery, Division of Critical Care Medicine, the Division of Infectious Diseases, the Department of Pharmacology, the Department of Computer Science and the Department of Healthcare Quality at Changhua Christian Hospital who were extremely helpful and provided invaluable assistance and support. The authors would also like to thank AllBio Co. Ltd. (Taichung, Taiwan) and Sengenics Corporation Pte Ltd. (Singapore) for technical support. This manuscript was edited by Editage Editing.

## Author contributions

**Acquisition of data**: CHC, ILC, HCY, and SHW

**Analysis and/or interpretation of data**: CHC, SCL, and CCH

**Approval of the version of the manuscript to be published**: CHC, ILC, HCY, SHW, SCL, and CCH

**Conception and design of study**: CHC, ILC, HCY, SHW, JSL, and CCH

**Drafting the manuscript**: CHC, and CCH

**Revising the manuscript critically for important intellectual content**: CHC, and CCH

## Supplementary Material

Supplemental Digital Content

## Supplementary Material

Supplemental Digital Content

## Supplementary Material

Supplemental Digital Content

## Supplementary Material

Supplemental Digital Content

## Supplementary Material

Supplemental Digital Content

## Supplementary Material

Supplemental Digital Content

## Supplementary Material

Supplemental Digital Content

## Supplementary Material

Supplemental Digital Content

## Supplementary Material

Supplemental Digital Content
