# Supplementary material for: Potential novel proteomic biomarkers for diagnosis of vertebral osteomyelitis identified using an immunomics protein array technique: Two cases reports
Source: Medicine (Baltimore). 2020 Oct 23;99(43):e22852. doi: 10.1097/MD.0000000000022852 (PMC7581026; doi:10.1097/MD.0000000000022852)
Supplement: Supplemental Digital Content [file medi-99-e22852-s001.docx]

Appendix 1

Biomarkers of vertebral osteomyelitis identified from literature

| **Year** | **Country** | **Author** | **Biomarker** | **Reference** |
| --- | --- | --- | --- | --- |
| 2013 | NY, US | Gedbjerg N | Anti-Glucosaminidase IgG | DOI: 10.2106/JBJS.L.01654 |
| 2015 | NY, US | Nishitani K | iron-regulated surface determinant protein B | doi: 10.1007/s11999-015-4354-2. |
| 2017 | Tianjin, China | Chunmiao Cui | procalcitonin | doi: 10.12659/MSM.904276 |
| 2017 | Puducherry, India | Karthikeyan Maharajan | procalcitonin | doi: 10.1186/1749-799X-8-19 |
| 2017 | Dresden, Germany | Sigrun Ruth Hofmann | CCL11/Eotaxin  Interleukin-6 | doi: 10.3389/fped.2017.00256 |
| 2017 | Hebei, China | Qingzhu Kong | matrix metalloproteinase-1 | doi: 10.1097/MD.0000000000004969 |
