# Supplementary material for: Potential novel proteomic biomarkers for diagnosis of vertebral osteomyelitis identified using an immunomics protein array technique: Two cases reports
Source: Medicine (Baltimore). 2020 Oct 23;99(43):e22852. doi: 10.1097/MD.0000000000022852 (PMC7581026; doi:10.1097/MD.0000000000022852)
Supplement: Supplemental Digital Content [file medi-99-e22852-s003.docx]

Appendix 3

Method for detecting biomarkers of vertebral osteomyelitis (Part A) and Annotation of top 10 shortlisted antigens (Part B) from the Immunome™ protein microarray platform with significant autoantibody responses in VO patients’ samples vs non-VO control.

(Part A)

Method for detecting biomarkers of vertebral osteomyelitis

Materials and Methods

Ethics statement

This study was approved by the Ethics Committee of the Changhua Christian Hospital (CCH IRB No. 180905) to allow the collection of nasal samples. Each participant provided written informed consent under a protocol that was approved by the institutional review board, and all methods were performed in accordance with these guidelines.

Setting and study design

The population living in the rural areas of Central Taiwan is mostly served by the Changhua Christian Hospital System (CCHS), which consists of 4000 beds. The Changhua Christian Hospital (CCH) is the largest among nine branch hospitals in the CCHS, and acts as an 1800-bed tertiary referral medical center situated in Central Taiwan. This study was conducted in the CCHS and was approved by the institutional review board of CCH. Subjects of VO, according to Zimmerli et al.^(1)^, were enrolled after signing written informed consent documents, and a case-control prospective study was conducted between July 1, 2018 and December 31, 2018 in the CCHS. Each subject had a medical chart, which contained medical diagnoses, surgical interventions, and other key information from the medical records. A standardized case report form was used to collect data on age, sex, site of involvement, underlying disease, clinical presentation, length of symptoms, diagnostic laboratory and radiographic reports (including MRI), medical treatment, interventions, and outcomes. Concomitant diseases included hypertension, chronic kidney disease, and diabetes. Mortality attributable to VO was also analyzed. A follow-up record was added from the date of diagnosis to the date of death, or to the date of the last available record.

Microbiological evaluation, serological examinations, and image examinations

Blood samples were routinely sent for tests for complete whole blood cell counts. Blood cells were cultured in the BD BACTEC FX (Becton Dickinson, Sparks, MD, USA) culture system, and identified using matrix-assisted laser desorption ionization-time of flight mass spectrometry (BioMerieux, Hazlewood, Mo.). The serologic examinations were performed as follows: C-reactive protein using near infrared particle immunoassay rate methodology (normal range, ＜0.748 mg/dL), and procalcitonin using electrochemiluminescence immunoassay (normal range, ＜0.5 ng/mL). The gold standard of VO diagnosis is MRI, used in the current study, according to Zimmerli et al.^(1)^.

Bioinformatics Methodology

The Immunome^TM^ Protein Assay protocol is shown at website of Sengenics (http://www.sengenics.com/wp-content/uploads/2019/07/Sengenics-KREX-User-Manual-June-2019.pdf). In brief, the penetrance-based fold change analysis was conducted between VO patients and negative and healthy controls to identify highly expressed proteins in each case sample. A step-by-step description of this method entails the following:

Step 1: Individual fold changes for both case and control were calculated by dividing the RFU value for each protein in each sample, H, by the mean of the RFU values of each protein across all control samples (i.e. background threshold).

Equation 1 Individual FC=HCase or Controlμ(HControl)

Step 2: For proteins with individual fold change of less than 2-fold above the background threshold, their signal intensities (RFU) were replaced with zeroes.

Step 3: Penetrance frequency (number of case and control samples with individual fold changes ≥ 2-fold) for both case (frequency case) groups was determined for each protein.

Equation 2 FrequencyCase=n(Individual FC (Case)) ≥2)

Equation 3 FrequencyControl=n(Individual FC (Control)) ≥2)

Step 4: Penetrance fold changes for both case and control groups were calculated for each protein.

Equation 4 Penetrance fold Changecase=μ(HControl[i])/ μ(HControl)

[𝑖]=HCase with FC Case ≧2 fold

An unsupervised clustering was performed for the following biomarkers across patients, controls, and pooled normal samples using a hierarchical clustering method, i.e. Ward’s method ^(2)^ with distance calculated based on Euclidean distance. The heatmap were plotted using ComplexHeatmap v 1.20.0 package ^(3)^ in Bioconductor.

Quality report

The Sengenics Immunome^TM^ Protein Array platform was utilized for high-throughput quantification of autoantibodies in five plasma samples collected from VO subjects, one non-VO control (bone-disease patient without infection), and one healthy control (healthy person). These were analyzed together with two pooled normal serum samples (Sengenics Internal quality control samples). All samples passed the quality control parameters that evaluated quantitative metrics related to the array and assay quality, as well as the consistency of the results. The results showed exceptional data consistency, which is measured as a percentage of the coefficient of variance (CV%). The intra-protein, intra-slide, and inter-array CV% for all proteins and control probes was below the quality control limit of 15%.

Study protocol


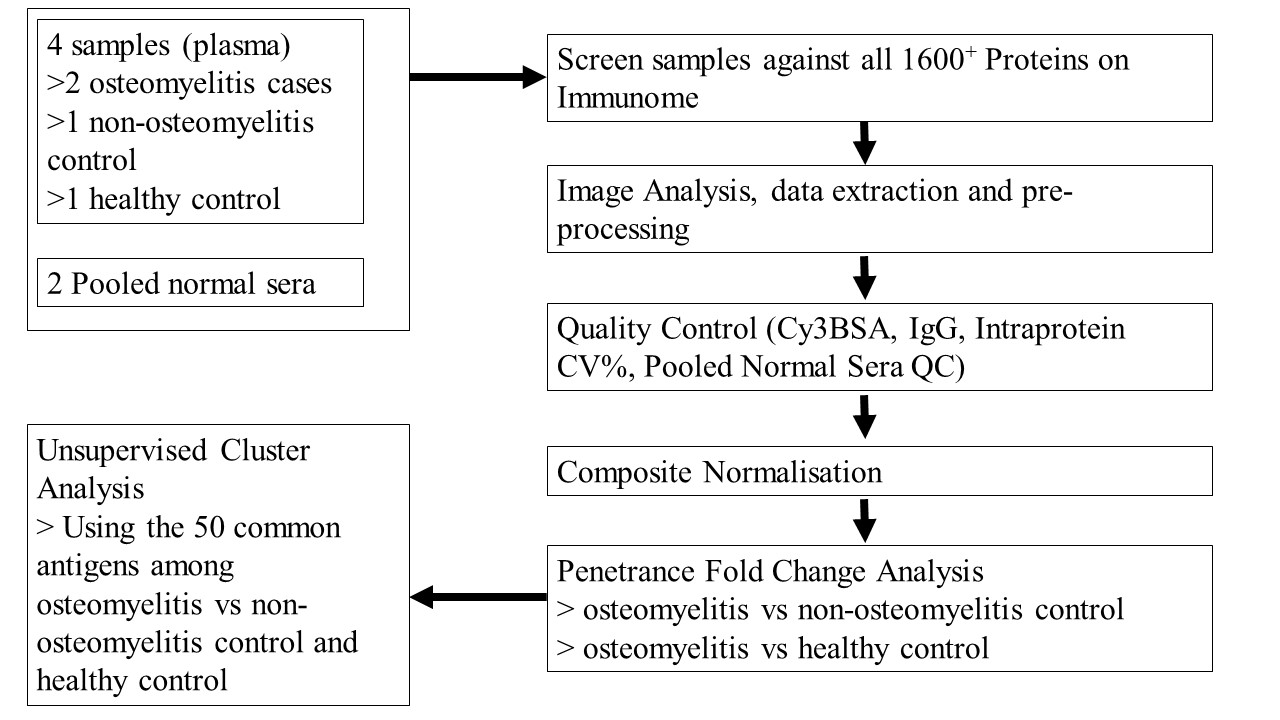


[References]

1. Zimmerli W. Osteomyelitis. In: Jameson JL, Fauci AS, Kasper DL, Hauser SL, Longo DL, Loscalzo J, editors. Harrison's Principles of Internal Medicine, 20e. New York, NY: McGraw-Hill Education; 2018.

2. Ward Jr JH. Hierarchical grouping to optimize an objective function. J Am Stat Assoc. 1963;58:236-44.

3. Gu Z, Eils R, Schlesner M. Complex heatmaps reveal patterns and correlations in multidimensional genomic data. Bioinformatics. 2016;32:2847-9.

(Part B)

Annotation of top 10 shortlisted antigens from the Immunome™ protein microarray platform with significant autoantibody responses in VO patients’ samples vs non-VO control.

| Protein Symbol | Protein Name | function |
| --- | --- | --- |
| **GGPS1** | Geranylgeranyl pyrophosphate synthase | Geranylgeranyl pyrophosphate synthase  Catalyzes the trans-addition of the three molecules of IPP onto DMAPP to form geranylgeranyl pyrophosphate, an important precursor of carotenoids and geranylated proteins. |
| **RPA2** | Replication protein A 32 kDa subunit | As part of the heterotrimeric replication protein A complex (RPA/RP-A), binds and stabilizes single-stranded DNA intermediates, that form during DNA replication or upon DNA stress. It prevents their reannealing and in parallel, recruits and activates different proteins and complexes involved in DNA metabolism. Thereby, it plays an essential role both in DNA replication and the cellular response to DNA damage. In the cellular response to DNA damage, the RPA complex controls DNA repair and DNA damage checkpoint activation. Through recruitment of ATRIP activates the ATR kinase a master regulator of the DNA damage response. It is required for the recruitment of the DNA double-strand break repair factors RAD51 and RAD52 to chromatin in response to DNA damage. Also recruits to sites of DNA damage proteins like XPA and XPG that are involved in nucleotide excision repair and is required for this mechanism of DNA repair. Plays also a role in base excision repair (BER) probably through interaction with UNG. Also recruits SMARCAL1/HARP, which is involved in replication fork restart, to sites of DNA damage. May also play a role in telomere maintenance |
| **SSNA1** | Sjoegren syndrome nuclear autoantigen 1 | identical protein binding. And ciliary basal body-plasma membrane docking ciliary receptor clustering involved in smoothened signaling pathway G2/M transition of mitotic cell cycle intraciliary transport regulation of G2/M transition of mitotic cell cycle |
| **ODC1** | Ornithine decarboxylase | Catalyzes the first and rate-limiting step of polyamine biosynthesis that converts ornithine into putrescine, which is the precursor for the polyamines, spermidine and spermine. Polyamines are essential for cell proliferation and are implicated in cellular processes, ranging from DNA replication to apoptosis |
| **KRT8** | Keratin, type II cytoskeletal 8 | Together with KRT19, helps to link the contractile apparatus to dystrophin at the costameres of striated muscle |
| **CRYAB** | Alpha-crystallin B chain | May contribute to the transparency and refractive index of the lens. Has chaperone-like activity, preventing aggregation of various proteins under a wide range of stress conditions. |
| **KRT19** | Keratin, type I cytoskeletal 19 | Involved in the organization of myofibers. Together with KRT8, helps to link the contractile apparatus to dystrophin at the costameres of striated muscle. |
| **ALDOA** | Fructose-bisphosphate aldolase A | Plays a key role in glycolysis and gluconeogenesis. In addition, may also function as scaffolding protein (By similarity). |
| **CRISP2** | Cysteine-rich secretory protein 2 | May regulate some ion channels' activity and therebye regulate calcium fluxes during sperm capacitation. |
| **PRKAR1A** | cAMP-dependent protein kinase type I-alpha regulatory subunit | Regulatory subunit of the cAMP-dependent protein kinases involved in cAMP signaling in cells. |
