# Supplementary material for: Potential novel proteomic biomarkers for diagnosis of vertebral osteomyelitis identified using an immunomics protein array technique: Two cases reports
Source: Medicine (Baltimore). 2020 Oct 23;99(43):e22852. doi: 10.1097/MD.0000000000022852 (PMC7581026; doi:10.1097/MD.0000000000022852)
Supplement: Supplemental Digital Content [file medi-99-e22852-s004.docx]

Appendix 4 Summary of clinical data from 4 subjects, including two patients information

| Variable | Subject One | Subject Two | Subject Three | Subject Four |
| --- | --- | --- | --- | --- |
| Group | Clinical Definitive Bone infection case | Clinical Definitive Bone infection case | Bone infection Negative-control | Healthy control |
| Demography | | | | |
| Age | 60 | 71 | 40 | 53 |
| Gender | Male | Female | Male | Male |
| Underlying diseases | | | | |
|  | End stage renal diseases | N | Type 2 diabetes mellitus | N |
|  | Type 2 diabetes mellitus |  | Hepatitis B |  |
|  | Hypertension |  |  |  |
| Initial Clinical presentation | | | | |
| Fever | Y | Y | N | N |
| Arthralgia | Y | Y | Y | N |
| Pain | Y | Y | Y | N |
| Initial laboratory data | | | | |
| Neutrophils (×1000/mm3), | 36.7 | 9.3 | 5.2 | 7.5 |
| Hb (g/dL) | 9.7 | 12.2 | 11.9 | 12.9 |
| Platelets (×1000/mm3) | 339 | 166 | 205 | 205 |
| ESR (mm/hr) | 32 | 86 | 58 | 15 |
| C-reactive protein (mg/L) | 3.48 | 0.28 | 0.13 | <0.748 |
| Procalcitonin (ng/mL) | 0.6 | 0.03 | 0.03 | 0.03 |
| Underlying bone condition | | | | |
| Image | MRI C/W Vertebral osteomyelitis | MRI, C/W Vertebral osteomyelitis |  |  |
| Microbiological data | | | | |
| Positive blood culture | *Staphylococcus aureus* | *Staphylococcus aureus* | N | N |
| Sampling Day | D5 | D4 | D4 | D6 |
| Surgical treatment | N | N | N | N |
| Medical treatment (final regimen) | | | | |
|  | Vancomycin with daptomycin | Oxacillin | No antibiotics | N |
| Outcome | Survive | Survive | Survive | Survive |

Note 1 MRI: Magnetic Resonance Imaging; C/W: compatible with
