# Supplementary material for: Potential novel proteomic biomarkers for diagnosis of vertebral osteomyelitis identified using an immunomics protein array technique: Two cases reports
Source: Medicine (Baltimore). 2020 Oct 23;99(43):e22852. doi: 10.1097/MD.0000000000022852 (PMC7581026; doi:10.1097/MD.0000000000022852)
Supplement: Supplemental Digital Content [file medi-99-e22852-s005.docx]

Appendix 5. List of top 10 shortlisted antigens from the Immunome™ protein microarray platform with significant autoantibody responses in BI patients’ samples vs negative control.

| Rank | Protein Symbol | Protein Name | Uniprot Accession | Penetrance Frequency (BI) | Penetrance Frequency % (BI) | Penetrance Fold Change (BI) | Mean (Negative Control) |
| --- | --- | --- | --- | --- | --- | --- | --- |
| 1 | **GGPS1** | Geranylgeranyl pyrophosphate synthase | O95749 | 2 | 100 | 20.078 | 71.795 |
| 2 | **RPA2** | Replication protein A 32 kDa subunit | P15927 | 2 | 100 | 18.884 | 93.522 |
| 3 | **SSNA1** | Sjoegren syndrome nuclear autoantigen 1 | O43805 | 2 | 100 | 17.281 | 75.573 |
| 4 | **ODC1** | Ornithine decarboxylase | P11926 | 2 | 100 | 14.832 | 59.986 |
| 5 | **KRT8** | Keratin, type II cytoskeletal 8 | P05787 | 2 | 100 | 14.481 | 87.382 |
| 6 | **CRYAB** | Alpha-crystallin B chain | P02511 | 2 | 100 | 14.137 | 110.762 |
| 7 | **KRT19** | Keratin, type I cytoskeletal 19 | P08727 | 2 | 100 | 14.026 | 158.704 |
| 8 | **ALDOA** | Fructose-bisphosphate aldolase A | P04075 | 2 | 100 | 13.233 | 77.699 |
| 9 | **CRISP2** | Cysteine-rich secretory protein 2 | P16562 | 2 | 100 | 12.781 | 88.09 |
| 10 | **PRKAR1A** | cAMP-dependent protein kinase type I-alpha regulatory subunit | P10644 | 2 | 100 | 12.677 | 76.99 |
