# Supplementary material for: Potential novel proteomic biomarkers for diagnosis of vertebral osteomyelitis identified using an immunomics protein array technique: Two cases reports
Source: Medicine (Baltimore). 2020 Oct 23;99(43):e22852. doi: 10.1097/MD.0000000000022852 (PMC7581026; doi:10.1097/MD.0000000000022852)
Supplement: Supplemental Digital Content [file medi-99-e22852-s006.docx]

Appendix 6　List of top 10 shortlisted antigens from the Immunome™ protein microarray platform with significant autoantibody responses in BI patients’ samples vs healthy control.

| Rank | Protein Symbol | Protein Name | UniprotAccession | Penetrance Frequency (BI) | Penetrance Frequency % (BI) | Penetrance Fold Change (BI) | Mean (Healthy Control) |
| --- | --- | --- | --- | --- | --- | --- | --- |
| 1 | **ZC4H2** | Zinc finger C4H2 domain-containing protein | Q9NQZ6 | 2 | 100 | 11.391 | 116.092 |
| 2 | **KRT8** | Keratin, type II cytoskeletal 8 | P05787 | 2 | 100 | 8.819 | 143.488 |
| 3 | **TACC1** | Transforming acidic coiled-coil-containing protein 1 | O75410 | 2 | 100 | 8.61 | 299.453 |
| 4 | **ODC1** | Ornithine decarboxylase | P11926 | 2 | 100 | 6.949 | 128.027 |
| 5 | **MOB3A** | MOB kinase activator 3A | Q96BX8 | 2 | 100 | 6.291 | 185.531 |
| 6 | **PCBD1** | Pterin-4-alpha-carbinolamine dehydratase | P61457 | 2 | 100 | 6.286 | 187.429 |
| 7 | **ALDOA** | Fructose-bisphosphate aldolase A | P04075 | 2 | 100 | 6.027 | 170.612 |
| 8 | **YWHAG** | 14-3-3 protein gamma | P61981 | 2 | 100 | 5.998 | 141.589 |
| 9 | **TK1** | Thymidine kinase, cytosolic | P04183 | 2 | 100 | 5.752 | 211.028 |
| 10 | **DNAJB1** | DnaJ homolog subfamily B member 1 | P25685 | 2 | 100 | 5.685 | 151.083 |
