# Supplementary material for: Potential novel proteomic biomarkers for diagnosis of vertebral osteomyelitis identified using an immunomics protein array technique: Two cases reports
Source: Medicine (Baltimore). 2020 Oct 23;99(43):e22852. doi: 10.1097/MD.0000000000022852 (PMC7581026; doi:10.1097/MD.0000000000022852)
Supplement: Supplemental Digital Content [file medi-99-e22852-s007.docx]

Appendix 7

Annotation of top 10 shortlisted antigens from the Immunome™ protein microarray platform with significant autoantibody responses in BI patients’ samples vs healthy control.

| Protein Symbol | Protein Name | function |
| --- | --- | --- |
| **ZC4H2** | Zinc finger C4H2 domain-containing protein | Plays a role in interneurons differentiation (PubMed:26056227). Involved in neuronal development and in neuromuscular junction formation. |
| **KRT8** | Keratin, type II cytoskeletal 8 | Together with KRT19, helps to link the contractile apparatus to dystrophin at the costameres of striated muscle |
| **TACC1** | Transforming acidic coiled-coil-containing protein 1 | Involved in transcription regulation induced by nuclear receptors, including in T3 thyroid hormone and all-trans retinoic acid pathways (PubMed:20078863). Might promote the nuclear localization of the receptors (PubMed:20078863). Likely involved in the processes that promote cell division prior to the formation of differentiated tissues |
| **ODC1** | Ornithine decarboxylase | Catalyzes the first and rate-limiting step of polyamine biosynthesis that converts ornithine into putrescine, which is the precursor for the polyamines, spermidine and spermine. Polyamines are essential for cell proliferation and are implicated in cellular processes, ranging from DNA replication to apoptosis. |
| **MOB3A** | MOB kinase activator 3A | May regulate the activity of kinases. |
| **PCBD1** | Pterin-4-alpha-carbinolamine dehydratase | Involved in tetrahydrobiopterin biosynthesis. Seems to both prevent the formation of 7-pterins and accelerate the formation of quinonoid-BH2. Coactivator for HNF1A-dependent transcription. Regulates the dimerization of homeodomain protein HNF1A and enhances its transcriptional activity. |
| **ALDOA** | Fructose-bisphosphate aldolase A | Plays a key role in glycolysis and gluconeogenesis. In addition, may also function as scaffolding protein (By similarity). |
| **YWHAG** | 14-3-3 protein gamma | Adapter protein implicated in the regulation of a large spectrum of both general and specialized signaling pathways. Binds to a large number of partners, usually by recognition of a phosphoserine or phosphothreonine motif. Binding generally results in the modulation of the activity of the binding partner |
| **TK1** | Thymidine kinase, cytosolic | Two forms have been identified in animal cells, one in cytosol and one in mitochondria. Activity of the cytosolic enzyme is high in proliferating cells and peaks during the S-phase of the cell cycle; it is very low in resting cells. |
| **DNAJB1** | DnaJ homolog subfamily B member 1 | As a co-chaperone for HSPA5 it is required for proper folding, trafficking or degradation of proteins (PubMed:10827079, PubMed:15525676, PubMed:29706351). Binds directly to both unfolded proteins that are substrates for ERAD and nascent unfolded peptide chains, but dissociates from the HSPA5-unfolded protein complex before folding is completed (PubMed:15525676). May help recruiting HSPA5 and other chaperones to the substrate. Stimulates HSPA5 ATPase activity (PubMed:10827079). It is necessary for maturation and correct trafficking of PKD1 (PubMed:29706351) |
