# Supplementary material for: Potential novel proteomic biomarkers for diagnosis of vertebral osteomyelitis identified using an immunomics protein array technique: Two cases reports
Source: Medicine (Baltimore). 2020 Oct 23;99(43):e22852. doi: 10.1097/MD.0000000000022852 (PMC7581026; doi:10.1097/MD.0000000000022852)
Supplement: Supplemental Digital Content [file medi-99-e22852-s008.docx]

Appendix 8 Disease associations of biomarkers identified across 5 plasma samples. Data was obtained from Open Targets platform.

| Disease full name | No. of associated targets | All targets |
| --- | --- | --- |
| Bone Disease | 24 | **MATN3, TP53, NPM1, PRDX1, GFAP, P3H4, IMPDH1, NCOA5, MIF, PRC1, EEF1D, MPZL2, PKNOX1, GNAO1, KRT8, LY86, PCBD1, TFG, CRADD** |
| Rare Genetic Bone Development Disorder | 18 | **MATN3, TP53, NPM1, P3H4, GFAP, PRDX1, NCOA5, IMPDH1, MIF, PRC1, MPZL2, PKNOX1, GNAO1, KRT8, LY86, PCBD1, ZC4H2, YWHAG** |
| Rare Genetic Bone Disease | 18 | **MATN3, TP53, NPM1, P3H4, GFAP, PRDX1, IMPDH1, NCOA5, MIF, PRC1, MPZL2, PKNOX1, GNAO1, KRT8, LY86, PCBD1, ZC4H2, YWHAG** |
| Aggrecan-Related Bone Disorder | 1 | **MATN3** |
| Bone Fracture | 4 | **MATN3, LY86, PRC1, PKNOX1** |
| Pulmonary Fibrosis And/Or Bone Marrow Failure, Telomere-Related, 1 | 1 | **TP53** |
| Femoral Neck Bone Mineral Density | 1 | **MEF2C** |
| Congenital Disorder Of Glycosylation-Related Bone Disorder | 2 | **TP53, PRDX1** |
| Rare Bone Disease Related To A Common Gene Or Pathway Defect | 3 | **MATN3, TP53, PRDX1** |
| Hip Bone Mineral Density | 1 | **MEF2C** |
| Type 11 Collagen-Related Bone Disorder | 1 | **MATN3** |
| Bone Measurement | 3 | **CRADD, MEF2C, LY86** |
| Bone Density | 2 | **MEF2C, CRADD** |
| Bone Fracture Related Measurement | 2 | **MEF2C, CRADD** |
| Type 2 Collagen-Related Bone Disorder | 1 | **MATN3** |
| Sulfation-Related Bone Disorder | 1 | **MATN3** |
| TRPV4-Related Bone Disorder | 1 | **TP53** |
| Heel Bone Mineral Density | 1 | **CRADD** |
| Primary Bone Dysplasia with Defective Bone Mineralization | 1 | **P3H4** |
