# Supplementary material for: Potential novel proteomic biomarkers for diagnosis of vertebral osteomyelitis identified using an immunomics protein array technique: Two cases reports
Source: Medicine (Baltimore). 2020 Oct 23;99(43):e22852. doi: 10.1097/MD.0000000000022852 (PMC7581026; doi:10.1097/MD.0000000000022852)
Supplement: Supplemental Digital Content [file medi-99-e22852-s009.docx]

Appendix 9

Annotation of biomarkers identified and obtained from Open Targets platform.

1

Bone Disease

| Protein Symbol | Protein Name | function |
| --- | --- | --- |
| **MATN3** | Matrilin-3 | Major component of the extracellular matrix of cartilage and may play a role in the formation of extracellular filamentous networks. |
| **TP53** | EKC/KEOPS complex subunit TP53RK | Component of the EKC/KEOPS complex that is required for the formation of a threonylcarbamoyl group on adenosine at position 37 (t6A37) in tRNAs that read codons beginning with adenine (PubMed:22912744, PubMed:27903914). The complex is probably involved in the transfer of the threonylcarbamoyl moiety of threonylcarbamoyl-AMP (TC-AMP) to the N6 group of A37 (PubMed:22912744, PubMed:27903914). TP53RK has ATPase activity in the context of the EKC/KEOPS complex and likely plays a supporting role to the catalytic subunit OSGEP (By similarity). Atypical protein kinase that phosphorylates 'Ser-15' of p53/TP53 protein and may therefore participate in its activation (PubMed:11546806) |
| **NPM1** | Nucleophosmin | Involved in diverse cellular processes such as ribosome biogenesis, centrosome duplication, protein chaperoning, histone assembly, cell proliferation, and regulation of tumor suppressors p53/TP53 and ARF. Binds ribosome presumably to drive ribosome nuclear export. Associated with nucleolar ribonucleoprotein structures and bind single-stranded nucleic acids. Acts as a chaperonin for the core histones H3, H2B and H4. Stimulates APEX1 endonuclease activity on apurinic/apyrimidinic (AP) double-stranded DNA but inhibits APEX1 endonuclease activity on AP single-stranded RNA. May exert a control of APEX1 endonuclease activity within nucleoli devoted to repair AP on rDNA and the removal of oxidized rRNA molecules. In concert with BRCA2, regulates centrosome duplication. Regulates centriole duplication: phosphorylation by PLK2 is able to trigger centriole replication. Negatively regulates the activation of EIF2AK2/PKR and suppresses apoptosis through inhibition of EIF2AK2/PKR autophosphorylation. Antagonizes the inhibitory effect of ATF5 on cell proliferation and relieves ATF5-induced G2/M blockade (PubMed:22528486). In complex with MYC enhances the transcription of MYC target genes (PubMed:25956029) |
| **PRDX1** | Peroxiredoxin-1 | 1  Thiol-specific peroxidase that catalyzes the reduction of hydrogen peroxide and organic hydroperoxides to water and alcohols, respectively. Plays a role in cell protection against oxidative stress by detoxifying peroxides and as sensor of hydrogen peroxide-mediated signaling events. Might participate in the signaling cascades of growth factors and tumor necrosis factor-alpha by regulating the intracellular concentrations of H2O2 (PubMed:9497357). Reduces an intramolecular disulfide bond in GDPD5 that gates the ability to GDPD5 to drive postmitotic motor neuron differentiation (By similarity).  2  The active site is a conserved redox-active cysteine residue, the peroxidatic cysteine (C(P)), which makes the nucleophilic attack on the peroxide substrate. The peroxide oxidizes the C(P)-SH to cysteine sulfenic acid (C(P)-SOH), which then reacts with another cysteine residue, the resolving cysteine (C(R)), to form a disulfide bridge. The disulfide is subsequently reduced by an appropriate electron donor to complete the catalytic cycle. In this typical 2-Cys peroxiredoxin, C(R) is provided by the other dimeric subunit to form an intersubunit disulfide. The disulfide is subsequently reduced by thioredoxin. |
| **GFAP** | Glial fibrillary acidic protein | GFAP, a class-III intermediate filament, is a cell-specific marker that, during the development of the central nervous system, distinguishes astrocytes from other glial cells. |
| **P3H4** | Endoplasmic reticulum protein SC65 | Part of a complex composed of PLOD1, P3H3 and P3H4 that catalyzes hydroxylation of lysine residues in collagen alpha chains and is required for normal assembly and cross-linking of collagen fibrils. Required for normal bone density and normal skin stability via its role in hydroxylation of lysine residues in collagen alpha chains and in collagen fibril assembly. |
| **IMPDH1** | Inosine-5'-monophosphate dehydrogenase 1 | 1  Catalyzes the conversion of inosine 5'-phosphate (IMP) to xanthosine 5'-phosphate (XMP), the first committed and rate-limiting step in the de novo synthesis of guanine nucleotides, and therefore plays an important role in the regulation of cell growth. Could also have a single-stranded nucleic acid-binding activity and could play a role in RNA and/or DNA metabolism. It may also have a role in the development of malignancy and the growth progression of some tumors.  2  Because IMPDH activity is tightly linked with cell proliferation, it has been recognized as a target for cancer and viral chemotherapy and as a target for immunosuppressive drugs. The activities of the antitumor drug tiazofurin, the antiviral drug ribavirin, and the immunosuppressive drugs mizoribine and mycophenolic acid (MPA) are attributed to the inhibition of IMPDH. In addition, bacterial and parasitic IMPDH's differ significantly from mammalian enzymes, which makes it a suitable target for anti-infective drugs. |
| **NCOA5** | Nuclear receptor coactivator 5 | Nuclear receptor coregulator that can have both coactivator and corepressor functions. Interacts with nuclear receptors for steroids (ESR1 and ESR2) independently of the steroid binding domain (AF-2) of the ESR receptors, and with the orphan nuclear receptor NR1D2. Involved in the coactivation of nuclear steroid receptors (ER) as well as the corepression of MYC in response to 17-beta-estradiol (E2). |
| **MIF** | Macrophage migration inhibitory factor | 1  Pro-inflammatory cytokine. Involved in the innate immune response to bacterial pathogens. The expression of MIF at sites of inflammation suggests a role as mediator in regulating the function of macrophages in host defense. Counteracts the anti-inflammatory activity of glucocorticoids. Has phenylpyruvate tautomerase and dopachrome tautomerase activity (in vitro), but the physiological substrate is not known. It is not clear whether the tautomerase activity has any physiological relevance, and whether it is important for cytokine activity.  2  Serum levels of MIF are elevated in patients with severe sepsis or septic shock. High levels of MIF are correlated with low survival. Drugs that inhibit tautomerase activity protect against death due to sepsis. |
| **PRC1** | Protein regulator of cytokinesis 1 | Key regulator of cytokinesis that cross-links antiparrallel microtubules at an average distance of 35 nM. Essential for controlling the spatiotemporal formation of the midzone and successful cytokinesis. Required for KIF14 localization to the central spindle and midbody. Required to recruit PLK1 to the spindle. Stimulates PLK1 phosphorylation of RACGAP1 to allow recruitment of ECT2 to the central spindle. Acts as an oncogene for promoting bladder cancer cells proliferation, apoptosis inhibition and carcinogenic progression (PubMed:17409436) |
| **EEF1D** | Elongation factor 1-delta | Isoform 1: EF-1-beta and EF-1-delta stimulate the exchange of GDP bound to EF-1-alpha to GTP, regenerating EF-1-alpha for another round of transfer of aminoacyl-tRNAs to the ribosome.  Isoform 2: Regulates induction of heat-shock-responsive genes through association with heat shock transcription factors and direct DNA-binding at heat shock promoter elements (HSE). |
| **MPZL2** | Myelin protein zero-like protein 2 | Mediates homophilic cell-cell adhesion. |
| **PKNOX1** | Homeobox protein PKNOX1 | Activates transcription in the presence of PBX1A and HOXA1 |
| **GNAO1** | Guanine nucleotide-binding protein G(o) subunit alpha | Guanine nucleotide-binding proteins (G proteins) are involved as modulators or transducers in various transmembrane signaling systems. The G(o) protein function is not clear. Stimulated by RGS14. |
| **KRT8** | Keratin, type II cytoskeletal 8 | 1  Together with KRT19, helps to link the contractile apparatus to dystrophin at the costameres of striated muscle  2  There are two types of cytoskeletal and microfibrillar keratin: I (acidic; 40-55 kDa) and II (neutral to basic; 56-70 kDa). |
| **LY86** | Lymphocyte antigen 86 | May cooperate with CD180 and TLR4 to mediate the innate immune response to bacterial lipopolysaccharide (LPS) and cytokine production. Important for efficient CD180 cell surface expression (By similarity) |
| **PCBD1** | Pterin-4-alpha-carbinolamine dehydratase | Involved in tetrahydrobiopterin biosynthesis. Seems to both prevent the formation of 7-pterins and accelerate the formation of quinonoid-BH2. Coactivator for HNF1A-dependent transcription. Regulates the dimerization of homeodomain protein HNF1A and enhances its transcriptional activity. |
| **TFG** | Protein TFG | Plays a role in the normal dynamic function of the endoplasmic reticulum (ER) and its associated microtubules (PubMed:23479643, PubMed:27813252). Required for secretory cargo traffic from the endoplasmic reticulum to the Golgi apparatus (PubMed:21478858). |
| **CRADD** | Death domain-containing protein CRADD | Adapter protein that associates with PIDD1 and the caspase CASP2 to form the PIDDosome, a complex that activates CASP2 and triggers apoptosis (PubMed:9044836, PubMed:15073321, PubMed:16652156, PubMed:17159900, PubMed:17289572). Also recruits CASP2 to the TNFR-1 signaling complex through its interaction with RIPK1 and TRADD and may play a role in the tumor necrosis factor-mediated signaling pathway (PubMed:8985253) |

2

Rare Genetic Bone Development Disorder

| Protein Symbol | Protein Name | function |
| --- | --- | --- |
| **GFAP** | Glial fibrillary acidic protein | GFAP, a class-III intermediate filament, is a cell-specific marker that, during the development of the central nervous system, distinguishes astrocytes from other glial cells. |
| **GNAO1** | Guanine nucleotide-binding protein G(o) subunit alpha | Guanine nucleotide-binding proteins (G proteins) are involved as modulators or transducers in various transmembrane signaling systems. The G(o) protein function is not clear. Stimulated by RGS14. |
| **IMPDH1** | Inosine-5'-monophosphate dehydrogenase 1 | 1  Catalyzes the conversion of inosine 5'-phosphate (IMP) to xanthosine 5'-phosphate (XMP), the first committed and rate-limiting step in the de novo synthesis of guanine nucleotides, and therefore plays an important role in the regulation of cell growth. Could also have a single-stranded nucleic acid-binding activity and could play a role in RNA and/or DNA metabolism. It may also have a role in the development of malignancy and the growth progression of some tumors.  2  Because IMPDH activity is tightly linked with cell proliferation, it has been recognized as a target for cancer and viral chemotherapy and as a target for immunosuppressive drugs. The activities of the antitumor drug tiazofurin, the antiviral drug ribavirin, and the immunosuppressive drugs mizoribine and mycophenolic acid (MPA) are attributed to the inhibition of IMPDH. In addition, bacterial and parasitic IMPDH's differ significantly from mammalian enzymes, which makes it a suitable target for anti-infective drugs. |
| **KRT8** | Keratin, type II cytoskeletal 8 | 1  Together with KRT19, helps to link the contractile apparatus to dystrophin at the costameres of striated muscle  2  There are two types of cytoskeletal and microfibrillar keratin: I (acidic; 40-55 kDa) and II (neutral to basic; 56-70 kDa). |
| **LY86** | Lymphocyte antigen 86 | May cooperate with CD180 and TLR4 to mediate the innate immune response to bacterial lipopolysaccharide (LPS) and cytokine production. Important for efficient CD180 cell surface expression (By similarity) |
| **MATN3** | Matrilin-3 | Major component of the extracellular matrix of cartilage and may play a role in the formation of extracellular filamentous networks. |
| **MIF** | Macrophage migration inhibitory factor | 1  Pro-inflammatory cytokine. Involved in the innate immune response to bacterial pathogens. The expression of MIF at sites of inflammation suggests a role as mediator in regulating the function of macrophages in host defense. Counteracts the anti-inflammatory activity of glucocorticoids. Has phenylpyruvate tautomerase and dopachrome tautomerase activity (in vitro), but the physiological substrate is not known. It is not clear whether the tautomerase activity has any physiological relevance, and whether it is important for cytokine activity.  2  Serum levels of MIF are elevated in patients with severe sepsis or septic shock. High levels of MIF are correlated with low survival. Drugs that inhibit tautomerase activity protect against death due to sepsis. |
| **MPZL2** | Myelin protein zero-like protein 2 | Mediates homophilic cell-cell adhesion. |
| **NCOA5** | Nuclear receptor coactivator 5 | Nuclear receptor coregulator that can have both coactivator and corepressor functions. Interacts with nuclear receptors for steroids (ESR1 and ESR2) independently of the steroid binding domain (AF-2) of the ESR receptors, and with the orphan nuclear receptor NR1D2. Involved in the coactivation of nuclear steroid receptors (ER) as well as the corepression of MYC in response to 17-beta-estradiol (E2). |
| **NPM1** | Nucleophosmin | Involved in diverse cellular processes such as ribosome biogenesis, centrosome duplication, protein chaperoning, histone assembly, cell proliferation, and regulation of tumor suppressors p53/TP53 and ARF. Binds ribosome presumably to drive ribosome nuclear export. Associated with nucleolar ribonucleoprotein structures and bind single-stranded nucleic acids. Acts as a chaperonin for the core histones H3, H2B and H4. Stimulates APEX1 endonuclease activity on apurinic/apyrimidinic (AP) double-stranded DNA but inhibits APEX1 endonuclease activity on AP single-stranded RNA. May exert a control of APEX1 endonuclease activity within nucleoli devoted to repair AP on rDNA and the removal of oxidized rRNA molecules. In concert with BRCA2, regulates centrosome duplication. Regulates centriole duplication: phosphorylation by PLK2 is able to trigger centriole replication. Negatively regulates the activation of EIF2AK2/PKR and suppresses apoptosis through inhibition of EIF2AK2/PKR autophosphorylation. Antagonizes the inhibitory effect of ATF5 on cell proliferation and relieves ATF5-induced G2/M blockade (PubMed:22528486). In complex with MYC enhances the transcription of MYC target genes (PubMed:25956029) |
| **P3H4** | Endoplasmic reticulum protein SC65 | Part of a complex composed of PLOD1, P3H3 and P3H4 that catalyzes hydroxylation of lysine residues in collagen alpha chains and is required for normal assembly and cross-linking of collagen fibrils. Required for normal bone density and normal skin stability via its role in hydroxylation of lysine residues in collagen alpha chains and in collagen fibril assembly. |
| **PCBD1** | Pterin-4-alpha-carbinolamine dehydratase | Involved in tetrahydrobiopterin biosynthesis. Seems to both prevent the formation of 7-pterins and accelerate the formation of quinonoid-BH2. Coactivator for HNF1A-dependent transcription. Regulates the dimerization of homeodomain protein HNF1A and enhances its transcriptional activity. |
| **PKNOX1** | Homeobox protein PKNOX1 | Activates transcription in the presence of PBX1A and HOXA1 |
| **PRC1** | Protein regulator of cytokinesis 1 | Key regulator of cytokinesis that cross-links antiparrallel microtubules at an average distance of 35 nM. Essential for controlling the spatiotemporal formation of the midzone and successful cytokinesis. Required for KIF14 localization to the central spindle and midbody. Required to recruit PLK1 to the spindle. Stimulates PLK1 phosphorylation of RACGAP1 to allow recruitment of ECT2 to the central spindle. Acts as an oncogene for promoting bladder cancer cells proliferation, apoptosis inhibition and carcinogenic progression (PubMed:17409436) |
| **PRDX1** | Peroxiredoxin-1 | 1  Thiol-specific peroxidase that catalyzes the reduction of hydrogen peroxide and organic hydroperoxides to water and alcohols, respectively. Plays a role in cell protection against oxidative stress by detoxifying peroxides and as sensor of hydrogen peroxide-mediated signaling events. Might participate in the signaling cascades of growth factors and tumor necrosis factor-alpha by regulating the intracellular concentrations of H2O2 (PubMed:9497357). Reduces an intramolecular disulfide bond in GDPD5 that gates the ability to GDPD5 to drive postmitotic motor neuron differentiation (By similarity).  2  The active site is a conserved redox-active cysteine residue, the peroxidatic cysteine (C(P)), which makes the nucleophilic attack on the peroxide substrate. The peroxide oxidizes the C(P)-SH to cysteine sulfenic acid (C(P)-SOH), which then reacts with another cysteine residue, the resolving cysteine (C(R)), to form a disulfide bridge. The disulfide is subsequently reduced by an appropriate electron donor to complete the catalytic cycle. In this typical 2-Cys peroxiredoxin, C(R) is provided by the other dimeric subunit to form an intersubunit disulfide. The disulfide is subsequently reduced by thioredoxin. |
| **TP53** | EKC/KEOPS complex subunit TP53RK | Component of the EKC/KEOPS complex that is required for the formation of a threonylcarbamoyl group on adenosine at position 37 (t6A37) in tRNAs that read codons beginning with adenine (PubMed:22912744, PubMed:27903914). The complex is probably involved in the transfer of the threonylcarbamoyl moiety of threonylcarbamoyl-AMP (TC-AMP) to the N6 group of A37 (PubMed:22912744, PubMed:27903914). TP53RK has ATPase activity in the context of the EKC/KEOPS complex and likely plays a supporting role to the catalytic subunit OSGEP (By similarity). Atypical protein kinase that phosphorylates 'Ser-15' of p53/TP53 protein and may therefore participate in its activation (PubMed:11546806) |
| **YWHAG** | YWHAG | Adapter protein implicated in the regulation of a large spectrum of both general and specialized signaling pathways. Binds to a large number of partners, usually by recognition of a phosphoserine or phosphothreonine motif. Binding generally results in the modulation of the activity of the binding partner |
| **ZC4H2** | ZC4H2 | Plays a role in interneurons differentiation (PubMed:26056227). Involved in neuronal development and in neuromuscular junction formation |

3

Rare Genetic Bone Disease

| Protein Symbol | Protein Name | function |
| --- | --- | --- |
| **GFAP** | Glial fibrillary acidic protein | GFAP, a class-III intermediate filament, is a cell-specific marker that, during the development of the central nervous system, distinguishes astrocytes from other glial cells. |
| **GNAO1** | Guanine nucleotide-binding protein G(o) subunit alpha | Guanine nucleotide-binding proteins (G proteins) are involved as modulators or transducers in various transmembrane signaling systems. The G(o) protein function is not clear. Stimulated by RGS14. |
| **IMPDH1** | Inosine-5'-monophosphate dehydrogenase 1 | 1  Catalyzes the conversion of inosine 5'-phosphate (IMP) to xanthosine 5'-phosphate (XMP), the first committed and rate-limiting step in the de novo synthesis of guanine nucleotides, and therefore plays an important role in the regulation of cell growth. Could also have a single-stranded nucleic acid-binding activity and could play a role in RNA and/or DNA metabolism. It may also have a role in the development of malignancy and the growth progression of some tumors.  2  Because IMPDH activity is tightly linked with cell proliferation, it has been recognized as a target for cancer and viral chemotherapy and as a target for immunosuppressive drugs. The activities of the antitumor drug tiazofurin, the antiviral drug ribavirin, and the immunosuppressive drugs mizoribine and mycophenolic acid (MPA) are attributed to the inhibition of IMPDH. In addition, bacterial and parasitic IMPDH's differ significantly from mammalian enzymes, which makes it a suitable target for anti-infective drugs. |
| **KRT8** | Keratin, type II cytoskeletal 8 | 1  Together with KRT19, helps to link the contractile apparatus to dystrophin at the costameres of striated muscle  2  There are two types of cytoskeletal and microfibrillar keratin: I (acidic; 40-55 kDa) and II (neutral to basic; 56-70 kDa). |
| **LY86** | Lymphocyte antigen 86 | May cooperate with CD180 and TLR4 to mediate the innate immune response to bacterial lipopolysaccharide (LPS) and cytokine production. Important for efficient CD180 cell surface expression (By similarity) |
| **MATN3** | Matrilin-3 | Major component of the extracellular matrix of cartilage and may play a role in the formation of extracellular filamentous networks. |
| **MIF** | Macrophage migration inhibitory factor | 1  Pro-inflammatory cytokine. Involved in the innate immune response to bacterial pathogens. The expression of MIF at sites of inflammation suggests a role as mediator in regulating the function of macrophages in host defense. Counteracts the anti-inflammatory activity of glucocorticoids. Has phenylpyruvate tautomerase and dopachrome tautomerase activity (in vitro), but the physiological substrate is not known. It is not clear whether the tautomerase activity has any physiological relevance, and whether it is important for cytokine activity.  2  Serum levels of MIF are elevated in patients with severe sepsis or septic shock. High levels of MIF are correlated with low survival. Drugs that inhibit tautomerase activity protect against death due to sepsis. |
| **MPZL2** | Myelin protein zero-like protein 2 | Mediates homophilic cell-cell adhesion. |
| **NCOA5** | Nuclear receptor coactivator 5 | Nuclear receptor coregulator that can have both coactivator and corepressor functions. Interacts with nuclear receptors for steroids (ESR1 and ESR2) independently of the steroid binding domain (AF-2) of the ESR receptors, and with the orphan nuclear receptor NR1D2. Involved in the coactivation of nuclear steroid receptors (ER) as well as the corepression of MYC in response to 17-beta-estradiol (E2). |
| **NPM1** | Nucleophosmin | Involved in diverse cellular processes such as ribosome biogenesis, centrosome duplication, protein chaperoning, histone assembly, cell proliferation, and regulation of tumor suppressors p53/TP53 and ARF. Binds ribosome presumably to drive ribosome nuclear export. Associated with nucleolar ribonucleoprotein structures and bind single-stranded nucleic acids. Acts as a chaperonin for the core histones H3, H2B and H4. Stimulates APEX1 endonuclease activity on apurinic/apyrimidinic (AP) double-stranded DNA but inhibits APEX1 endonuclease activity on AP single-stranded RNA. May exert a control of APEX1 endonuclease activity within nucleoli devoted to repair AP on rDNA and the removal of oxidized rRNA molecules. In concert with BRCA2, regulates centrosome duplication. Regulates centriole duplication: phosphorylation by PLK2 is able to trigger centriole replication. Negatively regulates the activation of EIF2AK2/PKR and suppresses apoptosis through inhibition of EIF2AK2/PKR autophosphorylation. Antagonizes the inhibitory effect of ATF5 on cell proliferation and relieves ATF5-induced G2/M blockade (PubMed:22528486). In complex with MYC enhances the transcription of MYC target genes (PubMed:25956029) |
| **P3H4** | Endoplasmic reticulum protein SC65 | Part of a complex composed of PLOD1, P3H3 and P3H4 that catalyzes hydroxylation of lysine residues in collagen alpha chains and is required for normal assembly and cross-linking of collagen fibrils. Required for normal bone density and normal skin stability via its role in hydroxylation of lysine residues in collagen alpha chains and in collagen fibril assembly. |
| **PCBD1** | Pterin-4-alpha-carbinolamine dehydratase | Involved in tetrahydrobiopterin biosynthesis. Seems to both prevent the formation of 7-pterins and accelerate the formation of quinonoid-BH2. Coactivator for HNF1A-dependent transcription. Regulates the dimerization of homeodomain protein HNF1A and enhances its transcriptional activity. |
| **PKNOX1** | Homeobox protein PKNOX1 | Activates transcription in the presence of PBX1A and HOXA1 |
| **PRC1** | Protein regulator of cytokinesis 1 | Key regulator of cytokinesis that cross-links antiparrallel microtubules at an average distance of 35 nM. Essential for controlling the spatiotemporal formation of the midzone and successful cytokinesis. Required for KIF14 localization to the central spindle and midbody. Required to recruit PLK1 to the spindle. Stimulates PLK1 phosphorylation of RACGAP1 to allow recruitment of ECT2 to the central spindle. Acts as an oncogene for promoting bladder cancer cells proliferation, apoptosis inhibition and carcinogenic progression (PubMed:17409436) |
| **PRDX1** | Peroxiredoxin-1 | 1  Thiol-specific peroxidase that catalyzes the reduction of hydrogen peroxide and organic hydroperoxides to water and alcohols, respectively. Plays a role in cell protection against oxidative stress by detoxifying peroxides and as sensor of hydrogen peroxide-mediated signaling events. Might participate in the signaling cascades of growth factors and tumor necrosis factor-alpha by regulating the intracellular concentrations of H2O2 (PubMed:9497357). Reduces an intramolecular disulfide bond in GDPD5 that gates the ability to GDPD5 to drive postmitotic motor neuron differentiation (By similarity).  2  The active site is a conserved redox-active cysteine residue, the peroxidatic cysteine (C(P)), which makes the nucleophilic attack on the peroxide substrate. The peroxide oxidizes the C(P)-SH to cysteine sulfenic acid (C(P)-SOH), which then reacts with another cysteine residue, the resolving cysteine (C(R)), to form a disulfide bridge. The disulfide is subsequently reduced by an appropriate electron donor to complete the catalytic cycle. In this typical 2-Cys peroxiredoxin, C(R) is provided by the other dimeric subunit to form an intersubunit disulfide. The disulfide is subsequently reduced by thioredoxin. |
| **TP53** | EKC/KEOPS complex subunit TP53RK | Component of the EKC/KEOPS complex that is required for the formation of a threonylcarbamoyl group on adenosine at position 37 (t6A37) in tRNAs that read codons beginning with adenine (PubMed:22912744, PubMed:27903914). The complex is probably involved in the transfer of the threonylcarbamoyl moiety of threonylcarbamoyl-AMP (TC-AMP) to the N6 group of A37 (PubMed:22912744, PubMed:27903914). TP53RK has ATPase activity in the context of the EKC/KEOPS complex and likely plays a supporting role to the catalytic subunit OSGEP (By similarity). Atypical protein kinase that phosphorylates 'Ser-15' of p53/TP53 protein and may therefore participate in its activation (PubMed:11546806) |
| **YWHAG** | YWHAG | Adapter protein implicated in the regulation of a large spectrum of both general and specialized signaling pathways. Binds to a large number of partners, usually by recognition of a phosphoserine or phosphothreonine motif. Binding generally results in the modulation of the activity of the binding partner |
| **ZC4H2** | ZC4H2 | Plays a role in interneurons differentiation (PubMed:26056227). Involved in neuronal development and in neuromuscular junction formation |

4

Aggrecan-Related Bone Disorder

| Protein Symbol | Protein Name | function |
| --- | --- | --- |
| **MATN3** | Matrilin-3 | Major component of the extracellular matrix of cartilage and may play a role in the formation of extracellular filamentous networks. |

5

Bone Fracture

| Protein Symbol | Protein Name | function |
| --- | --- | --- |
| **MATN3** | Matrilin-3 | Major component of the extracellular matrix of cartilage and may play a role in the formation of extracellular filamentous networks. |
| **LY86** | Lymphocyte antigen 86 | May cooperate with CD180 and TLR4 to mediate the innate immune response to bacterial lipopolysaccharide (LPS) and cytokine production. Important for efficient CD180 cell surface expression (By similarity) |
| **PKNOX1** | Homeobox protein PKNOX1 | Activates transcription in the presence of PBX1A and HOXA1 |
| **PRC1** | Protein regulator of cytokinesis 1 | Key regulator of cytokinesis that cross-links antiparrallel microtubules at an average distance of 35 nM. Essential for controlling the spatiotemporal formation of the midzone and successful cytokinesis. Required for KIF14 localization to the central spindle and midbody. Required to recruit PLK1 to the spindle. Stimulates PLK1 phosphorylation of RACGAP1 to allow recruitment of ECT2 to the central spindle. Acts as an oncogene for promoting bladder cancer cells proliferation, apoptosis inhibition and carcinogenic progression (PubMed:17409436) |

6

Pulmonary Fibrosis And/Or Bone Marrow Failure, Telomere-Related, 1

| Protein Symbol | Protein Name | function |
| --- | --- | --- |
| **TP53** | EKC/KEOPS complex subunit TP53RK | Component of the EKC/KEOPS complex that is required for the formation of a threonylcarbamoyl group on adenosine at position 37 (t6A37) in tRNAs that read codons beginning with adenine (PubMed:22912744, PubMed:27903914). The complex is probably involved in the transfer of the threonylcarbamoyl moiety of threonylcarbamoyl-AMP (TC-AMP) to the N6 group of A37 (PubMed:22912744, PubMed:27903914). TP53RK has ATPase activity in the context of the EKC/KEOPS complex and likely plays a supporting role to the catalytic subunit OSGEP (By similarity). Atypical protein kinase that phosphorylates 'Ser-15' of p53/TP53 protein and may therefore participate in its activation (PubMed:11546806) |

7

Femoral Neck Bone Mineral Density

| Protein Symbol | Protein Name | function |
| --- | --- | --- |
| **MEF2C** | Myocyte-specific enhancer factor 2C | Transcription activator which binds specifically to the MEF2 element present in the regulatory regions of many muscle-specific genes. Controls cardiac morphogenesis and myogenesis, and is also involved in vascular development. Enhances transcriptional activation mediated by SOX18. Plays an essential role in hippocampal-dependent learning and memory by suppressing the number of excitatory synapses and thus regulating basal and evoked synaptic transmission. Crucial for normal neuronal development, distribution, and electrical activity in the neocortex. Necessary for proper development of megakaryocytes and platelets and for bone marrow B-lymphopoiesis. Required for B-cell survival and proliferation in response to BCR stimulation, efficient IgG1 antibody responses to T-cell-dependent antigens and for normal induction of germinal center B-cells. May also be involved in neurogenesis and in the development of cortical architecture (By similarity). Isoforms that lack the repressor domain are more active than isoform 1 |

8

Congenital Disorder Of Glycosylation-Related Bone Disorder

| Protein Symbol | Protein Name | function |
| --- | --- | --- |
| **TP53** | EKC/KEOPS complex subunit TP53RK | Component of the EKC/KEOPS complex that is required for the formation of a threonylcarbamoyl group on adenosine at position 37 (t6A37) in tRNAs that read codons beginning with adenine (PubMed:22912744, PubMed:27903914). The complex is probably involved in the transfer of the threonylcarbamoyl moiety of threonylcarbamoyl-AMP (TC-AMP) to the N6 group of A37 (PubMed:22912744, PubMed:27903914). TP53RK has ATPase activity in the context of the EKC/KEOPS complex and likely plays a supporting role to the catalytic subunit OSGEP (By similarity). Atypical protein kinase that phosphorylates 'Ser-15' of p53/TP53 protein and may therefore participate in its activation (PubMed:11546806) |
| **PRDX1** | Peroxiredoxin-1 | 1  Thiol-specific peroxidase that catalyzes the reduction of hydrogen peroxide and organic hydroperoxides to water and alcohols, respectively. Plays a role in cell protection against oxidative stress by detoxifying peroxides and as sensor of hydrogen peroxide-mediated signaling events. Might participate in the signaling cascades of growth factors and tumor necrosis factor-alpha by regulating the intracellular concentrations of H2O2 (PubMed:9497357). Reduces an intramolecular disulfide bond in GDPD5 that gates the ability to GDPD5 to drive postmitotic motor neuron differentiation (By similarity).  2  The active site is a conserved redox-active cysteine residue, the peroxidatic cysteine (C(P)), which makes the nucleophilic attack on the peroxide substrate. The peroxide oxidizes the C(P)-SH to cysteine sulfenic acid (C(P)-SOH), which then reacts with another cysteine residue, the resolving cysteine (C(R)), to form a disulfide bridge. The disulfide is subsequently reduced by an appropriate electron donor to complete the catalytic cycle. In this typical 2-Cys peroxiredoxin, C(R) is provided by the other dimeric subunit to form an intersubunit disulfide. The disulfide is subsequently reduced by thioredoxin. |

9

Rare Bone Disease Related To A Common Gene Or Pathway Defect

| Protein Symbol | Protein Name | function |
| --- | --- | --- |
| **MATN3** | Matrilin-3 | Major component of the extracellular matrix of cartilage and may play a role in the formation of extracellular filamentous networks. |
| **TP53** | EKC/KEOPS complex subunit TP53RK | Component of the EKC/KEOPS complex that is required for the formation of a threonylcarbamoyl group on adenosine at position 37 (t6A37) in tRNAs that read codons beginning with adenine (PubMed:22912744, PubMed:27903914). The complex is probably involved in the transfer of the threonylcarbamoyl moiety of threonylcarbamoyl-AMP (TC-AMP) to the N6 group of A37 (PubMed:22912744, PubMed:27903914). TP53RK has ATPase activity in the context of the EKC/KEOPS complex and likely plays a supporting role to the catalytic subunit OSGEP (By similarity). Atypical protein kinase that phosphorylates 'Ser-15' of p53/TP53 protein and may therefore participate in its activation (PubMed:11546806) |
| **PRDX1** | Peroxiredoxin-1 | 1  Thiol-specific peroxidase that catalyzes the reduction of hydrogen peroxide and organic hydroperoxides to water and alcohols, respectively. Plays a role in cell protection against oxidative stress by detoxifying peroxides and as sensor of hydrogen peroxide-mediated signaling events. Might participate in the signaling cascades of growth factors and tumor necrosis factor-alpha by regulating the intracellular concentrations of H2O2 (PubMed:9497357). Reduces an intramolecular disulfide bond in GDPD5 that gates the ability to GDPD5 to drive postmitotic motor neuron differentiation (By similarity).  2  The active site is a conserved redox-active cysteine residue, the peroxidatic cysteine (C(P)), which makes the nucleophilic attack on the peroxide substrate. The peroxide oxidizes the C(P)-SH to cysteine sulfenic acid (C(P)-SOH), which then reacts with another cysteine residue, the resolving cysteine (C(R)), to form a disulfide bridge. The disulfide is subsequently reduced by an appropriate electron donor to complete the catalytic cycle. In this typical 2-Cys peroxiredoxin, C(R) is provided by the other dimeric subunit to form an intersubunit disulfide. The disulfide is subsequently reduced by thioredoxin. |

10

Hip Bone Mineral Density

| Protein Symbol | Protein Name | function |
| --- | --- | --- |
| **MEF2C** | Myocyte-specific enhancer factor 2C | Transcription activator which binds specifically to the MEF2 element present in the regulatory regions of many muscle-specific genes. Controls cardiac morphogenesis and myogenesis, and is also involved in vascular development. Enhances transcriptional activation mediated by SOX18. Plays an essential role in hippocampal-dependent learning and memory by suppressing the number of excitatory synapses and thus regulating basal and evoked synaptic transmission. Crucial for normal neuronal development, distribution, and electrical activity in the neocortex. Necessary for proper development of megakaryocytes and platelets and for bone marrow B-lymphopoiesis. Required for B-cell survival and proliferation in response to BCR stimulation, efficient IgG1 antibody responses to T-cell-dependent antigens and for normal induction of germinal center B-cells. May also be involved in neurogenesis and in the development of cortical architecture (By similarity). Isoforms that lack the repressor domain are more active than isoform 1 |

11

Type 11 Collagen-Related Bone Disorder

| Protein Symbol | Protein Name | function |
| --- | --- | --- |
| **MATN3** | Matrilin-3 | Major component of the extracellular matrix of cartilage and may play a role in the formation of extracellular filamentous networks. |

12

Bone Measurement

| Protein Symbol | Protein Name | function |
| --- | --- | --- |
| **CRADD** | Death domain-containing protein CRADD | Adapter protein that associates with PIDD1 and the caspase CASP2 to form the PIDDosome, a complex that activates CASP2 and triggers apoptosis (PubMed:9044836, PubMed:15073321, PubMed:16652156, PubMed:17159900, PubMed:17289572). Also recruits CASP2 to the TNFR-1 signaling complex through its interaction with RIPK1 and TRADD and may play a role in the tumor necrosis factor-mediated signaling pathway (PubMed:8985253) |
| **MEF2C** | Myocyte-specific enhancer factor 2C | Transcription activator which binds specifically to the MEF2 element present in the regulatory regions of many muscle-specific genes. Controls cardiac morphogenesis and myogenesis, and is also involved in vascular development. Enhances transcriptional activation mediated by SOX18. Plays an essential role in hippocampal-dependent learning and memory by suppressing the number of excitatory synapses and thus regulating basal and evoked synaptic transmission. Crucial for normal neuronal development, distribution, and electrical activity in the neocortex. Necessary for proper development of megakaryocytes and platelets and for bone marrow B-lymphopoiesis. Required for B-cell survival and proliferation in response to BCR stimulation, efficient IgG1 antibody responses to T-cell-dependent antigens and for normal induction of germinal center B-cells. May also be involved in neurogenesis and in the development of cortical architecture (By similarity). Isoforms that lack the repressor domain are more active than isoform 1 |
| **LY86** | Lymphocyte antigen 86 | May cooperate with CD180 and TLR4 to mediate the innate immune response to bacterial lipopolysaccharide (LPS) and cytokine production. Important for efficient CD180 cell surface expression (By similarity) |

13

Bone Density

| Protein Symbol | Protein Name | function |
| --- | --- | --- |
| **MEF2C** | Myocyte-specific enhancer factor 2C | Transcription activator which binds specifically to the MEF2 element present in the regulatory regions of many muscle-specific genes. Controls cardiac morphogenesis and myogenesis, and is also involved in vascular development. Enhances transcriptional activation mediated by SOX18. Plays an essential role in hippocampal-dependent learning and memory by suppressing the number of excitatory synapses and thus regulating basal and evoked synaptic transmission. Crucial for normal neuronal development, distribution, and electrical activity in the neocortex. Necessary for proper development of megakaryocytes and platelets and for bone marrow B-lymphopoiesis. Required for B-cell survival and proliferation in response to BCR stimulation, efficient IgG1 antibody responses to T-cell-dependent antigens and for normal induction of germinal center B-cells. May also be involved in neurogenesis and in the development of cortical architecture (By similarity). Isoforms that lack the repressor domain are more active than isoform 1 |
| **CRADD** | Death domain-containing protein CRADD | Adapter protein that associates with PIDD1 and the caspase CASP2 to form the PIDDosome, a complex that activates CASP2 and triggers apoptosis (PubMed:9044836, PubMed:15073321, PubMed:16652156, PubMed:17159900, PubMed:17289572). Also recruits CASP2 to the TNFR-1 signaling complex through its interaction with RIPK1 and TRADD and may play a role in the tumor necrosis factor-mediated signaling pathway (PubMed:8985253) |

14

Bone Fracture Related Measurement

| Protein Symbol | Protein Name | function |
| --- | --- | --- |
| **MEF2C** | Myocyte-specific enhancer factor 2C | Transcription activator which binds specifically to the MEF2 element present in the regulatory regions of many muscle-specific genes. Controls cardiac morphogenesis and myogenesis, and is also involved in vascular development. Enhances transcriptional activation mediated by SOX18. Plays an essential role in hippocampal-dependent learning and memory by suppressing the number of excitatory synapses and thus regulating basal and evoked synaptic transmission. Crucial for normal neuronal development, distribution, and electrical activity in the neocortex. Necessary for proper development of megakaryocytes and platelets and for bone marrow B-lymphopoiesis. Required for B-cell survival and proliferation in response to BCR stimulation, efficient IgG1 antibody responses to T-cell-dependent antigens and for normal induction of germinal center B-cells. May also be involved in neurogenesis and in the development of cortical architecture (By similarity). Isoforms that lack the repressor domain are more active than isoform 1 |
| **CRADD** | Death domain-containing protein CRADD | Adapter protein that associates with PIDD1 and the caspase CASP2 to form the PIDDosome, a complex that activates CASP2 and triggers apoptosis (PubMed:9044836, PubMed:15073321, PubMed:16652156, PubMed:17159900, PubMed:17289572). Also recruits CASP2 to the TNFR-1 signaling complex through its interaction with RIPK1 and TRADD and may play a role in the tumor necrosis factor-mediated signaling pathway (PubMed:8985253) |

15

Type 2 Collagen-Related Bone Disorder

| Protein Symbol | Protein Name | function |
| --- | --- | --- |
| **MATN3** | Matrilin-3 | Major component of the extracellular matrix of cartilage and may play a role in the formation of extracellular filamentous networks. |

16

Sulfation-Related Bone Disorder

| Protein Symbol | Protein Name | function |
| --- | --- | --- |
| **MATN3** | Matrilin-3 | Major component of the extracellular matrix of cartilage and may play a role in the formation of extracellular filamentous networks. |

17

TRPV4-Related Bone Disorder

| Protein Symbol | Protein Name | function |
| --- | --- | --- |
| **TP53** | EKC/KEOPS complex subunit TP53RK | Component of the EKC/KEOPS complex that is required for the formation of a threonylcarbamoyl group on adenosine at position 37 (t6A37) in tRNAs that read codons beginning with adenine (PubMed:22912744, PubMed:27903914). The complex is probably involved in the transfer of the threonylcarbamoyl moiety of threonylcarbamoyl-AMP (TC-AMP) to the N6 group of A37 (PubMed:22912744, PubMed:27903914). TP53RK has ATPase activity in the context of the EKC/KEOPS complex and likely plays a supporting role to the catalytic subunit OSGEP (By similarity). Atypical protein kinase that phosphorylates 'Ser-15' of p53/TP53 protein and may therefore participate in its activation (PubMed:11546806) |

18

Heel Bone Mineral Density

| Protein Symbol | Protein Name | function |
| --- | --- | --- |
| **CRADD** | Death domain-containing protein CRADD | Adapter protein that associates with PIDD1 and the caspase CASP2 to form the PIDDosome, a complex that activates CASP2 and triggers apoptosis (PubMed:9044836, PubMed:15073321, PubMed:16652156, PubMed:17159900, PubMed:17289572). Also recruits CASP2 to the TNFR-1 signaling complex through its interaction with RIPK1 and TRADD and may play a role in the tumor necrosis factor-mediated signaling pathway (PubMed:8985253) |

19

Primary Bone Dysplasia with Defective Bone Mineralization

| Protein Symbol | Protein Name | function |
| --- | --- | --- |
| **P3H4** | Endoplasmic reticulum protein SC65 | Part of a complex composed of PLOD1, P3H3 and P3H4 that catalyzes hydroxylation of lysine residues in collagen alpha chains and is required for normal assembly and cross-linking of collagen fibrils. Required for normal bone density and normal skin stability via its role in hydroxylation of lysine residues in collagen alpha chains and in collagen fibril assembly. |
